# Measuring Health-Related Quality of Life in Tuberous Sclerosis Complex – Psychometric Evaluation of Three Instruments in Individuals With Refractory Epilepsy

**DOI:** 10.3389/fphar.2018.00964

**Published:** 2018-08-30

**Authors:** Petrus J. de Vries, David N. Franz, Paolo Curatolo, Rima Nabbout, Maureen Neary, Fabian Herbst, Kate Sully, Elaine Brohan, Bryan Bennett, John A. Lawson

**Affiliations:** ^1^Division of Child and Adolescent Psychiatry, University of Cape Town, Cape Town, South Africa; ^2^Department of Pediatrics and Neurology, Cincinnati Children’s Hospital Medical Center, Cincinnati, OH, United States; ^3^Tor Vergata University Hospital, Rome, Italy; ^4^Hospital Necker-Enfants Malades, Paris Descartes University, Paris, France; ^5^Novartis Pharmaceuticals Corporation, East Hanover, NJ, United States; ^6^Novartis Pharma AG, Basel, Switzerland; ^7^Adelphi Values, Bollington, United Kingdom; ^8^Tuberous Sclerosis Multidisciplinary Management Clinic, Sydney Children’s Hospital, Randwick, NSW, Australia

**Keywords:** tuberous sclerosis complex, refractory epilepsy, health related quality of life, psychometric properties, QOLCE, QOLIE-AD-48, QOLIE-31-P

## Abstract

Tuberous sclerosis complex (TSC) is a rare genetic disease associated with significant disease burden and considerable impact on health-related quality of life (HRQL). Currently no disease-specific clinical outcome assessments evaluate HRQL in individuals with TSC. A multi-center phase III study EXIST-3 (NCT01713946) assessed the efficacy and safety of two trough exposure ranges (Low exposure, LE: 3–7 ng/mL and high exposure, HE: 9–15 ng/mL) of adjunctive everolimus in patients aged 2–65 years with TSC and refractory partial-onset seizures (*N* = 366). Three age-specific HRQL measures were included as secondary endpoints including: quality of life in childhood epilepsy (QOLCE; caregiver-report for aged 2- < 11), the Quality of Life in Epilepsy Inventory for Adolescents-48 (QOLIE-AD-48; self-report, aged ≥ 11- < 18), and the Quality of Life in Epilepsy Inventory-31-Problems (QOLIE-31-P; self-report, aged ≥ 18). Intellectual ability was evaluated using the Wechsler Non-Verbal (WNV) Scale of Ability. *Post hoc* analyses were performed on the core phase primary data from EXIST-3 to evaluate the psychometric properties of the HRQL measures and calculate meaningful change estimates. Results showed that a significant subset of the trial sample (4–21 year olds) scored in the intellectual disability range, as assessed by the WNV. Psychometric analyses of the three epilepsy measures (including reliability, validity, and ability to detect change) supported the appropriateness for use in TSC. Distribution-based meaningful change estimates were generated for each HRQL measure, with estimates for the QOLIE-31-P total score largely consistent with the published literature. To our knowledge, this is the first evaluation using clinical trial data to establish the psychometric properties of the QOLCE, QOLIE-AD-48, and QOLIE-31-P for use in individuals with TSC. These findings increase confidence in the measures as valid and reliable for use in clinical trials and future research in patients with TSC.

## Introduction

Tuberous sclerosis complex (TSC) is a rare genetic disease which causes benign tumors in many different organs (Henske et al., 2016). Neurological and TSC-Associated Neuropsychiatric Disorders (TAND) are associated with the greatest burden of disease (Curatolo et al., 2015; de Vries et al., 2015; Leclezio and de Vries, 2016). Reports from the patient perspective, identified through TSC patient and caregiver forums, underline the impact of TAND, in particular, on health-related quality of life (HRQL) with reports of time off work/school, emotional impacts such as depression and feelings of isolation, and cognitive impacts such as delays in speech and slow processing skills (Tuberous Sclerosis Alliance, 2017).

Although the disease burden associated with TSC is well-documented (Curatolo et al., 2015; de Vries et al., 2015; Leclezio and de Vries, 2016), there are currently no disease-specific clinical outcome assessments (COAs) that assess HRQL in individuals with TSC. This is most likely due to the relative rarity of the condition with a birth incidence around 1 in 6,000 live births (Osborne et al., 1991). Selecting the optimal COA measure to assess the individual experience of a rare condition can be challenging. In line with recommendations from the International Society For Pharmacoeconomics and Outcomes Research (ISPOR) Good Practices Task Force and European Organization for Rare Disorders (EURORDIS), existing item banks or COA measures on the target population or similar populations are considered a “*practical solution given the obstacles associated with the development of a de novo COA for use in Rare Disease populations*” (European Organisation for Rare Disorders, 2011; Benjamin et al., 2017).

The international, multi-center phase III study EXIST-3 (NCT01713946) assessed the efficacy and safety of two trough exposure ranges (Low exposure, LE: 3–7 ng/mL and high exposure, HE: 9–15 ng/mL) of adjunctive everolimus in patients aged 2–65 years with TSC and refractory partial-onset seizures (*N* = 366) (French et al., 2016). The trial included three age-specific patient-reported/observer-reported outcome (PRO/ObsRO) measures as secondary endpoints: the Quality of Life in Childhood Epilepsy (QOLCE) for individuals aged < 11 years (Sabaz et al., 2003), the Quality of Life in Epilepsy Inventory for Adolescents-48 (QOLIE-AD-48) for individuals aged 11 to < 18 years (Cramer et al., 1999), and the Quality of Life in Epilepsy Inventory-31-Problems (QOLIE-31-P) for individuals aged ≥ 18 years (Cramer et al., 1998). These measures were developed to assess HRQL in individuals with epilepsy. Even though these instruments were developed for individuals with a broad range of epilepsy syndromes rather than for TSC-specific epilepsy, comparison of the qualitative epilepsy literature (McEwan et al., 2004; Elliott et al., 2005) and TSC user/carer forums (Tuberous Sclerosis Alliance, 2017) confirms a clear conceptual overlap in the impacts reported by both groups and the items captured in these COAs. The measures have established psychometric properties including internal consistency, test–retest reliability, and construct validity in the general epilepsy population. However, the psychometric properties of these instruments have not been evaluated in relation to individuals with TSC with or without refractory seizures (Cramer et al., 1998, 1999; Sabaz et al., 2000).

Generating evidence of the psychometric properties of a COA measure in the relevant patient population is critical for its credibility and acceptance by stakeholders. In particular, regulatory agencies such as the European Medicines Agency (EMA) (European Medicines Agency, 2005) and the US Food and Drug Administration (FDA) (Food and Drug Administration, 2009), and clinicians who wish to use these measures in research or clinical practice. It is also important that HRQL data can be interpreted in the context of what level of change in scores over time is clinically meaningful (improvement or worsening). Currently, there are no clinically meaningful change estimates available for the QOLCE or QOLIE-AD-48 in any epilepsy-related population. Whilst there are clinically meaningful change estimates in the literature for the QOLIE-31-P (all assessed change over time within a group), these estimates were derived in individuals with refractory epilepsy, rather than in a TSC population (Wiebe et al., 2002; Cramer et al., 2004; Borghs et al., 2012).

Here, we used data from EXIST-3 to evaluate the psychometric properties of the QOLCE, QOLIE-AD-48, and QOLIE-31-P in a TSC population and to calculate meaningful change estimates for these three HRQL measures.

## Materials and Methods

### Clinical Trial Design

Full details of the clinical trial design (NCT01713946) are described elsewhere (French et al., 2016). In brief, EXIST-3 was a three-arm, randomized, multi-center (99 centers across 25 countries), double-blind, placebo-controlled phase III study. The study consisted of three phases: 8 week baseline phase, 18 week core phase and a ≥48 week extension phase (extension data not included here). The primary efficacy endpoint was change from baseline in seizure frequency for each of the two everolimus exposure ranges compared with placebo during the last 12 weeks of the core phase, defined as response rate by the EMA (reduction in seizure frequency) and median percentage reduction in seizure frequency by the FDA. HRQL and intellectual ability were analyzed as secondary endpoints, using the QOLCE, QOLIE-AD-48, or QOLIE-31-P and Wechsler Non-Verbal (WNV) Scale of Ability, respectively. Individuals with TSC or their caregivers, were required to complete the relevant age-specific HRQL measure (QOLCE; QOLIE-AD-48; QOLIE-31-P) and WNV were performed with individuals with TSC at baseline (week 0 – reflecting on 4 weeks prior to randomization) and at end of core phase (week 18 – reflecting on the last 4 weeks of core phase). The study also assessed safety, according to the National Cancer Institute Common Toxicity Criteria for Adverse Events version 4.03.

### Measures Used

In line with recommendations from the FDA PRO guidance (Food and Drug Administration, 2009), the EMA reflection paper (European Medicines Agency, 2005) and the ISPOR Good Research Practices for the Assessment of Children and Adolescence Task Force (Matza et al., 2013), age-specific HRQL measures were administered to the relevant age groups within the clinical trial.

The QOLCE is an ObsRO measure (parent/caregiver reports the signs/symptoms and functional impacts observed in their child), designed to assess HRQL in individuals aged 4 to 18 years old with epilepsy (Sabaz et al., 2000, 2003). For the purpose of the trial the QOLCE was completed by parents/caregivers of individuals with TSC aged < 11 years old at randomization. The measure contains 92 items across five age-relevant domains namely physical function (12 items), emotional well-being (19 items), cognitive function (23 items), social function (12 items) and behavior (23 items). The QOLCE includes two general health items and one overall QoL item. In line with the literature, the cognitive subscales were completed only for individuals aged ≥ 6 to < 11 years old in the trial (Sabaz et al., 2003).

The QOLIE-AD-48 is a self-report PRO measure designed to assess HRQL in individuals aged 11–18 years old with epilepsy (Cramer et al., 1999). For the purpose of the trial the QOLIE-AD-48 was administered to individuals aged 11 to < 18 years old. The measure contains 48 items across eight domains namely epilepsy impact (12 items), memory/concentration (10 items), attitudes toward epilepsy (4 items), physical functioning (5 items), stigma (6 items), social support (4 items), school behavior (4 items), and health perceptions (3 items).

The QOLIE-31-P is a self-report PRO measure designed to assess HRQL in adults aged > 18 years old with epilepsy (Cramer et al., 1998). For the purpose of the trial the QOLIE-31-P was therefore administered to individuals aged ≥ 18 years old. The measure contains 39 items, of which 30 are used to make up seven domains namely seizure worry (5 items), overall QoL (2 items), emotional well-being (5 items), energy/fatigue (4 items), cognitive (6 items), medication effects (3 items), and social function (5 items). The remaining nine items (a distress item for each domain, an overall health item and an item which ranks the importance of each domain), did not contribute to the total score.

For each of the three HRQL measures scores can range from 0 to 100, with higher scores indicating a greater level of functioning and HRQL.

The WNV is a non-verbal assessment of general ability, designed for individuals aged 4–21 years old from diverse cultural and linguistic groups, those with limited language skills and those with language disorders (Wechsler et al., 2006). The single ability score derived for the full four subtests can range from 30 to 170 and the individual subtest scores can range from 10 to 90. Children aged 4 to 7 years 11 months completed the matrices and recognition subtests of the WNV, and individuals aged 8 to 21 years 11 months old completed the matrices and spatial span subtests, to derive prorated intellectual ability scores.

### Psychometric Analyses

A range of psychometric analyses were performed *post hoc* on the core phase primary data (cutoff date October, 2015) from EXIST-3 to assess the measurement properties and generate meaningful change estimates for each of the three HRQL measures in patients with TSC and refractory partial-onset seizures. In line with best practice for evaluation of PROs for use in clinical trials of pharmaceutical products (Food and Drug Administration, 2009), three main domains of psychometric evaluation were selected – item-level and dimensionality analysis, scale-level analysis, and clinically meaningful change analysis. **Table 1** outlines the range of psychometric analyses performed in each of these domains. Psychometric analyses were performed for each HRQL measure using the full analysis set (FAS; i.e., patients aged < 11, 11 to < 18, and ≥ 18 years at randomization) who completed the specific HRQL measure at baseline (week 0) and at end of core phase (week 18). Where there were violations in normalcy the appropriate non-parametric equivalent test was conducted.

**Table 1:** Overview of psychometric analyses conducted.

Analysis	Description
**Item-level and dimensionality analyses**	
Floor and ceiling effects	• Floor and ceiling effects were assessed by examining the proportion of respondents endorsing the lowest (floor) and highest (ceiling) possible response option for each item of the three HRQL measures. • An item with a floor effect > 20% or a ceiling effect > 20% in the sample was flagged (Terwee et al., 2007)
Construct validity: item-convergent and discriminant validity	• Item to scale correlations were calculated to explore the dimensionality and factor structure of the respective measures. Two criteria were considered: ∘ Item convergent validity: the item-to-scale correlation coefficient between each item and domain should be > 0.40 or higher (Cappelleri et al., 2013) ∘ Item discriminant validity: each item should have a higher item-to-scale correlation coefficient with its own domain than with any other domain and show small (<0.40) or negligible correlations (Cappelleri et al., 2013)

**Scale-level analyses**	
Internal consistency	• Internal consistency reliability was evaluated using Cronbach’s alpha coefficient for all multi-item domains. • This criterion was considered to be met if the Cronbach’s alpha coefficient was ≥0.70 (Cronbach, 1951; Nunnally and Bernstein, 1994)
Known-groups validity	• Construct validity was assessed using the known-groups method (Hedges and Olkin, 1985) to evaluate differences in total scores among respondents who differ on health/disease related variable at baseline: ∘ Level of intellectual disability (two levels: <70 single ability composite WNV score and ≥70 single ability composite WNV score) • The criterion was considered to be met when significantly different total scores (defined as *p* < 0.05) were obtained between the defined subgroups (Hattie and Cooksey, 1984)
Ability to detect change	• *t*-Tests were used to examine the extent to which HRQL scores related to corresponding changes in partial-onset seizure frequency between the defined subgroups (Husted et al., 2000) • Patients were categorized as: ∘ Responders (at least a 50% reduction from baseline in seizure frequency) ∘ Non-responders (less than a 50% reduction from Baseline in seizure frequency)

**Clinically meaningful change analyses**	
Distribution-based analysis	• Distribution-based analyses were conducted to generate change estimates, including: calculation of 0.5 of a standard deviation (SD) and standard error of measurement (SEM) (Wyrwich et al., 1999; Norman et al., 2003; de Vet et al., 2006; Revicki et al., 2008)

## Results

### Completion Rates

At the end of core phase (week 18), the overall completion rate for the QOLCE (completed by caregivers) was high across treatment arms (84%, 166/197), compared to the lower completion rates for the patient self-report QOLIE-AD-48 (36%, 37/102) and QOLIE-31-P (49%, 33/67) (**Table 2**).

**Table 2:** Overall completion rates for each HRQL measure at end of core phase (week 18) across treatment arms.

HRQL measure	Total % (n/N)	LE (n/N)	HE (n/N)	Placebo (n/N)
QOLCE	84% (*N* = 166/197)	54/65	58/69	54/63
QOLIE-AD-48	36% (*N* = 37/102)	13/31	14/38	10/33
QOLIE-31-P	49% (*N* = 33/67)	10/21	11/23	12/23

*HE, high exposure; LE, low exposure.*

### Intellectual Disability Before Starting Everolimus

At baseline (week 0) 43% of children aged 4 to 7 years old scored in the intellectual disability range, 17% of children scored in the normal intellectual ability range and 40% had missing data (see **Table 3**). At baseline (week 0) 49.1% of individuals aged 8 to 21 years old scored in the intellectual disability range, 19.4% scored in the normal intellectual ability range and 31.5% had missing data.

**Table 3:** Baseline (week 0) level of intellectual ability based on WNV composite score by age group.

Age group (N)	Level of intellectual ability	*N* (%)
4–7 years old (*n* = 100)	Normal intellectual ability (WNV ≥ 70)	17 (17%)
	Intellectual disability (WNV < 70)	43 (43%)
	Missing data	40 (40%)
8–21 years old (*n* = 165)	Normal intellectual ability (WNV ≥ 70)	32 (19.4%)
	Intellectual disability (WNV < 70)	81 (49.1%)
	Missing data	52 (31.5%)

*WNV, Wechsler Non-Verbal.*

### Item-Level and Dimensionality Analyses

#### Floor and Ceiling Effects

Approximately 26% of QOLCE items across five subscales (physical restrictions, language, other cognitive, behavior, and attention/concentration subscales), demonstrated floor effects at baseline and at the end of the core phase (**Table 4**). Around 22% of QOLCE items across eight subscales (physical restrictions, depression, control/helplessness, anxiety, self-esteem, social interactions, stigma item, and behavior subscales) showed ceiling effects at baseline and end of core phase.

**Table 4:** Floor and ceiling effects at baseline (week 0) and end of core phase (week 18).

HRQL measure	Floor effects	Ceiling effects
QOLCE	**24/92 items (26%)** 7 items – ‘Physical restrictions’ 6 items – ‘Language’ 5 items – ‘Attention/concentration’	**20/92 items (22%)** 4 items – ‘Anxiety’ 4 items – ‘Behavior’
QOLIE-AD-48	**4/48 items (8%)** 4 items – ‘Attitudes toward epilepsy’	**37/48 items (77%)** Items were across 8/9 domains, except for the health perception domain.
QOLIE-31-P	**3/30 items (10%)** 1 item – ‘social function’ 2 items – ‘seizure worry’	**27/30 items (90%)** Items were across all seven domains

Around 8% of QOLIE-AD-48 items, in the attitudes toward epilepsy domain, showed floor effects at baseline and end of core phase. Conversely, 77% of items, across eight of the nine domains (except for the health perception domain) showed ceiling effects at baseline and end of core phase.

Around 10% of QOLIE-31-P items across two domains (social function and seizure worry) demonstrated floor effects at baseline and at the end of the core phase. Conversely, 90% of items, across all seven domains, showed ceiling effects at baseline and end of core phase.

### Construct Validity: Item-Convergent and Discriminant Validity

Multi-trait analyses (see **Table 1** for definition) at baseline and end of core phase demonstrated that for each of the HRQL measures, the majority of items were appropriately placed in the correct subscales, as they met the threshold for item convergent validity (*r* ≥ 0.40) and item discriminant validity criterion (*r* < 0.40) (Cappelleri et al., 2013).

**Table 5** shows the items that did not meet the criteria for item convergent validity. Item discriminant validity was met in all cases.

**Table 5:** Items with below threshold item-convergent validity at baseline (week 0) and end of core phase (week 18) for each HRQL measure.

HRQL Measure	Item convergent validity		Item discriminant validity
	Subscale/domain (*n* = items)	Correlation coefficient	
QOLCE	Behavior (*n* = 4)	0.158–0.377	All items met the criterion for discriminant validity
	Anxiety (*n* = 3)	0.047–0.399	
	Depression (*n* = 2)	0.045–0.340	
	Social interactions (*n* = 2)	0.054–0.248	
	Physical restrictions (*n* = 1)	0.128–0.194	
	Control/helplessness (*n* = 1)	0.069–0.266	
	Self-esteem (*n* = 1)	0.284–0.335	
QOLIE-AD-48	School behavior (*n* = 2)	0.241–0.395	All items met the criterion for discriminant validity
	Epilepsy impact (*n* = 1)	0.278–0.326	
	Physical functioning (*n* = 1)	0.333–0.386	
	Health perceptions (*n* = 1)	0.161–0.258	
QOLIE-31-P	Medication effects (*n* = 1)	0.294–0.324	All items met the criterion for discriminant validity
	Social function (*n* = 1)	0.283–0.311	

### Scale-Level Analyses

#### Internal Consistency Reliability

##### QOLCE

**Table 6** shows the internal consistency reliability for the QOLCE overall QoL score and subscale scores. The total score and subscales were ≥0.70, apart from the five gray shaded subscales on the table (self-esteem, depression, anxiety, control/helplessness, and social interactions subscales).

**Table 6:** Cronbach’s alpha for QOLCE at baseline (week 0) and end of core phase (week 18).

Domain	Cronbach’s alpha	
	Baseline	End of core phase
Overall QoL score	0.94	0.95
Memory	0.94	0.92
Language	0.91	0.93
Attention/concentration	0.86	0.85
Social activities	0.86	0.83
Behavior	0.81	0.85
Physical restrictions	0.76	0.78
Other cognitive	0.76	0.85
Energy/fatigue	0.72	0.71
Self-esteem	0.70	0.63
Anxiety	0.68	0.57
Social interactions	0.63	0.38
Control/helplessness	0.49	0.68
Depression	0.41	0.55

*Gray shaded cells indicate items in this domain that did not meet threshold of ≥0.70 Cronbach’s alpha coefficient.*

##### QOLIE-AD-48

**Table 7** shows the internal consistency reliability for the QOLIE-AD-48 total score and domain scores. The total score and all domains were ≥0.70, apart from the two gray shaded domains on the table (school behavior and health perceptions).

**Table 7:** Cronbach’s alpha for QOLIE-AD-48 at baseline (week 0) and end of core phase (week 18).

Domain	Cronbach’s alpha	
	Baseline	End of core phase
Total score	0.93	0.91
Memory/concentration	0.95	0.92
Epilepsy impact	0.90	0.91
Attitudes toward epilepsy	0.86	0.89
Social support	0.80	0.93
Stigma	0.80	0.84
School behavior	0.78	0.45
Physical functioning	0.72	0.76
Health perceptions	0.58	0.24

*Gray shaded cells indicate items in this domain that did not meet threshold of ≥0.70 Cronbach’s alpha coefficient.*

##### QOLIE-31-P

**Table 8** shows the internal consistency reliability for the QOLIE-31-P total score and domain scores. The total score and all domains were ≥0.70, apart from the gray shaded domain on the table (social function).

**Table 8:** Cronbach’s alpha for QOLIE-31-P at baseline (week 0) and end of core phase (week 18).

Domain	Cronbach’s alpha	
	Baseline	End of core phase
Total core	0.90	0.92
Emotional well-being	0.84	0.86
Seizure worry	0.83	0.81
Energy/fatigue	0.82	0.82
Cognitive	0.78	0.85
Overall quality of life	0.72	0.90
Medication effects	0.71	0.72
Social function	0.61	0.70

*Gray shaded cells indicate items in this domain that did not meet threshold of ≥0.70 Cronbach’s alpha coefficient.*

### Construct Validity: Known Groups Method

There was evidence of known groups validity in a TSC population using level of intellectual ability as a grouping variable (**Table 9**). Total scores on the QOLCE and QOLIE-AD-48 reported at baseline were able to discriminate between those individuals with or without intellectual disability, as categorized by single ability scores on the WNV. Individuals with normal intellectual ability demonstrated statistically significant higher overall HRQL scores at baseline, with a moderate effect size, on the QOLCE and QOLIE-AD-48 (non-significant trend). Construct validity for the QOLIE-31-P was not analyzed due to limitations in sample size.

**Table 9:** Difference in QOLCE and QOLIE-AD-48 total scores with known groups categorized by intellectual ability status at baseline (week 0).

HRQL measure	Group	Number	Mean scores *(SD)*	*t*-Test *p*-value	Effect size
QOLCE	WNV ≥ 70	32	55.9 (11.4)	0.007^∗∗^	−0.621
	WNV < 70	64	48.1 (13.8)		
QOLIE-AD-48	WNV ≥ 70	14	66.5 (14.3)	0.051	−0.685
	WNV < 70	26	55.8 (16.9)		

*<70 = intellectual disability, ≥70 = normal intellectual ability. QOLCE = completed by caregivers, QOLIE-AD-48 = completed by individuals with TSC, ^∗^p < 0.05, ^∗∗^p < 0.01, ^∗∗∗^p < 0.001.*

### Ability to Detect Change

**Tables 10**–**12** shows the extent to which the HRQL scores on each measure changed with a change (in either direction) in seizure response status. Mean change scores for the majority of domains across each of the measures were greatest for patients classified as ‘responders.’ Of note, 3/16 QOLCE subscales (depression, attention/concentration and social interactions) and 1/9 QOLIE-AD-48 domains (school behavior) did not follow this pattern.

**Table 10:** Change scores and standardized effect sizes on the QOLCE based on the change from baseline in seizure frequency during the last 12 weeks of the core phase.

Domain	Category	Mean change	*SD*	Effect size	*t*-Test *p*-value
Quality of life item	Responders	11.1	23.4	0.54	0.051
	Non-responders	3.1	24.7	0.14	
Physical restrictions	Responders	3.5	15.2	0.19	0.155
	Non-responders	0.2	11.2	0.01	
Energy/fatigue	Responders	3.2	16.6	0.16	0.387
	Non-responders	0.7	19.0	0.04	
Depression	Responders	−0.6	13.4	−0.04	0.719
	Non-responders	0.2	13.4	0.01	
Anxiety	Responders	1.9	15.7	0.11	0.326
	Non-responders	−0.9	15.1	−0.06	
Control/helplessness	Responders	3.5	20.1	0.18	0.192
	Non-responders	−1.1	18.9	−0.07	
Self-esteem	Responders	1.3	16.2	0.07	0.359
	Non-responders	−1.9	15.9	−0.12	
Memory	Responders	0.0	23.6	0.00	0.936
	Non-responders	−0.4	17.2	−0.01	
Attention/concentration	Responders	−0.7	26.2	−0.03	0.328
	Non-responders	3.6	19.8	0.16	
Language	Responders	4.3	22.1	0.20	0.329
	Non-responders	0.5	16.3	0.02	
Other cognitive	Responders	1.7	22.0	0.07	0.776
	Non-responders	0.5	22.2	0.02	
Social interactions	Responders	2.0	15.8	0.08	0.368
	Non-responders	5.3	20.5	0.24	
Social activities	Responders	7.5	26.6	0.22	0.427
	Non-responders	4.0	26.6	0.12	
Stigma item	Responders	17.2	32.3	0.53	0.072
	Non-responders	6.3	34.6	0.19	
Behavior	Responders	3.8	11.0	0.26	0.198
	Non-responders	1.2	11.7	0.07	
General health item	Responders	9.9	27.7	0.42	0.187
	Non-responders	4.2	26.9	0.15	
Overall quality of life score	Responders	5.8	11.0	0.39	0.016^∗^
	Non-responders	1.7	10.7	0.12	

*^∗^Items in this domain met significant level of p < 0.05 demonstrating a significant difference between responders vs. non-responders.*

**Table 11:** Change scores and standardized effect sizes on QOLIE-AD-48 based on the change from baseline in seizure frequency during the last 12 weeks of the core phase.

Domain	Category	Mean change	*SD*	Effect size	*t*-Test *p*-value
Health perceptions	Responders	13.5	15.8	0.62	0.060
	Non-responders	4.2	13.2	0.21	
Physical functioning	Responders	7.5	21.6	0.42	0.635
	Non-responders	3.8	22.9	0.13	
Epilepsy impact	Responders	11.9	23.0	0.79	0.152
	Non-responders	1.5	14.1	0.06	
Memory/concentration	Responders	4.7	14.6	0.22	0.489
	Non-responders	1.2	14.7	0.04	
School behavior	Responders	-2.9	12.1	-0.25	0.241
	Non-responders	4.6	21.0	0.22	
Social support	Responders	2.9	24.0	0.11	0.187
	Non-responders	-11.1	33.4	-0.52	
Stigma	Responders	5.6	20.9	0.26	0.496
	Non-responders	0.5	21.9	0.002	
Attitudes toward epilepsy	Responders	8.8	19.8	0.63	0.316
	Non-responders	1.8	20.7	0.08	
Total score	Responders	8.2	12.4	0.88	0.155
	Non-responders	2.6	10.7	0.14	

**Table 12:** Change scores and standardized effect sizes on QOLIE-31-P scores based on the change from baseline in seizure frequency during the last 12 weeks of the core phase.

Domain	Category	Mean change	*SD*	Effect size	*t*-Test *p*-value
Quality of life item	Responders	7.5	10.2	0.39	0.121
	Non-responders	−2.7	16.0	−0.18	
Emotional well-being	Responders	11.4	25.4	0.36	0.066
	Non-responders	−5.8	20.3	−0.43	
Social function	Responders	19.1	23.6	1.19	0.088
	Non-responders	2.3	22.2	0.10	
Cognitive	Responders	21.1	19.5	1.60	0.024^∗^
	Non-responders	1.8	19.2	0.08	
Medication effects	Responders	5.2	26.2	0.24	0.301
	Non-responders	−5.9	24.3	−0.22	
Seizure worry	Responders	18.3	25.7	0.65	0.019^∗^
	Non-responders	0.0	15.0	0.00	
Energy/fatigue	Responders	6.0	23.2	0.23	0.579
	Non-responders	1.0	20.0	0.06	
Total score	Responders	15.2	15.7	0.74	0.021^∗^
	Non-responders	−0.6	15.3	−0.03	

*^∗^Items in this domain met significant level of p < 0.05 demonstrating a significant difference between responders vs. non-responders.*

Differences in mean changes observed among patients classified as ‘responders’ compared to those classified as ‘non-responders’ were statistically significant (*p* < 0.05) for the QOLCE overall QoL score and the QOLIE-31-P total score.

### Clinically Meaningful Change Analysis

**Table 13** shows the results of the distribution-based analyses for each of the HRQL measures. Using the 0.5 SD criterion (Norman et al., 2003; de Vet et al., 2006), estimates range from 6.0 for the QOLCE, 8.1 for the QOLIE-AD-48 and 11.0 for the QOLIE-31-P. Lower estimates were derived using the SEM (Wyrwich et al., 1999; de Vet et al., 2006), which range from 2.9 for the QOLCE, 4.2 for the QOLIE-AD-48, to 7.1 for the QOLIE-31-P.

**Table 13:** Distribution-based methods: meaningful change estimates for QOLCE, QOLIE-AD-48, and QOLIE-31-P overall HRQL score.

HRQL measure	1/2 SD ^[1]^	Reliability ^[2]^	*SEM*
QOLCE	6.0	0.94	2.9
QOLIE-AD-48	8.1	0.93	4.2
QOLIE-31-P	11.0	0.90	7.1

*[1] 0.5 standard deviation estimate based upon the standard deviation at baseline. [2] Reliability is based on internal consistency reliability computed at baseline.*

## Discussion

When selecting an optimal COA measure to assess the patient experience of a rare condition, it is often necessary, and acceptable, to use an existing COA measure developed for a similar population (European Organisation for Rare Disorders, 2011; Benjamin et al., 2017). However, generating evidence of the psychometric properties of a COA measure in the relevant patient population is critical for acceptance by stakeholders, and may identify unique psychometric characteristics of importance for clinical practice or research in the specific rare disease (European Medicines Agency, 2005). To our knowledge, this is the first evaluation using clinical trial data to establish the psychometric properties of the QOLCE, QOLIE-AD-48, and QOLIE-31-P for use in individuals with TSC.

### Intellectual Disability

Our analysis demonstrated that a significant subset of the trial sample (4–21 year olds) scored in the intellectual disability range, as assessed by the WNV, an observation consistent with the wide distribution of intellectual ability in individuals with TSC (Curatolo et al., 2015; de Vries et al., 2015; Leclezio and de Vries, 2016). This may have had implications on individuals’ ability to self-report on their HRQL and may, in part, explain the low completion rates for the two self-report PRO measures (QOLIE-AD-48 and QOLIE-31-P). Cognitive interviews to establish the appropriateness of self-report, and level of concordance between patient and informant-report, would support the transition of these measures from self-report to informant-report, which may result in more complete data (Matza et al., 2013).

### Item Level Analysis

Investigation of item performance demonstrated ceiling effects for the majority of items on the QOLIE-AD-48 and QOLIE-31-P, indicating that a high proportion of patients were satisfied with their HRQL at baseline. As such, the amount of improvement possible is limited within domains in the QOLIE-AD-48 (8/9 domains, except for health perceptions) and the QOLIE-31-P (7/7 domains). These findings may suggest that in rare diseases such as TSC where a wide range of intellectual ability is observed, existing measures may not be sufficiently sensitive to capture change in HRQL at the ‘upper end’ of functioning. This may be a potential limitation and a potential avenue for future measure development.

### Scale Level Analysis

The results of the scale-level analyses indicate good to excellent internal consistency (Cronbach’s alpha) for each of the HRQL measures in a TSC population, at the total score level and largely at the domain level. In line with the original development papers for the QOLCE (Sabaz et al., 2003), QOLIE-AD-48 (Cramer et al., 1999), and QOLIE-31-P (Cramer et al., 1998) (generated in epilepsy sample) the alpha coefficient surpassed 0.70 at the total score level. Interestingly, on the QOLCE 4 out of 16 subscales did not demonstrate acceptable levels of internal consistency reliability (i.e., <0.70). It should be noted that these fours subscales contain six or fewer items, and Cronbach’s alpha tends to be lower with lower number of items (Streiner et al., 2015). In addition, these results may suggest that the items contained within the subscales are not one-dimensional, for example the control/helplessness scale which includes items about feeling in control, as well as feeling excited or interested in something.

Construct validity was assessed using the known groups method. The QOLCE and QOLIE-AD-48 were able to distinguish between individuals with and without intellectual disability. Individuals with normal intellectual ability demonstrated higher overall HRQL scores at baseline, on the QOLCE (significant difference) and QOLIE-AD-48 (non-significant trend). The results may suggest that individuals who were able to complete the measures had higher overall ability and associated HRQL. However, as the WNV assesses individuals aged from 4–7 years old and 8–21 years old, the latter group encompasses individuals whose caregivers completed the QOLCE on their behalf, as well as individuals who completed the QOLIE-AD-48, meaning that it is hard to link the WNV data to the age-specific HRQL measures.

The QOLCE, QOLIE-AD-48, and QOLIE-31-P were shown to be responsive to improvements in seizure frequency. Across all three measures mean change scores were greatest for individuals who demonstrated a greater reduction in seizure frequency. Confirming the ability of an instrument to detect change strengthens the rationale to conduct meaningful change analysis, which focuses on establishing a threshold for meaningful change for each instrument.

### Meaningful Change Analysis

As there were no appropriate external anchors (i.e., not sufficiently correlated to the HRQL scores), interpretation of change was only explored using distribution-based methods. Meaningful change estimates were generated for each HRQL measure, with estimates for the QOLIE-31-P total score largely in line with the literature, although domain estimates were higher than previously estimated (Wiebe et al., 2002; Cramer et al., 2004; Borghs et al., 2012). The variability in estimations of meaningful change may reflect differences in methodological approaches between the current study and previous work, i.e., distribution based vs. anchor approaches (previous work utilized a Patient Global Impression of Change item) and patient populations, i.e., epilepsy vs. TSC (Borghs et al., 2012). TSC is a very complex genetic disorder and the clinical disease spectrum is highly variable (Curatolo et al., 2015; de Vries et al., 2015; Leclezio and de Vries, 2016). Manifestations of the disorder can range from mild to profound intellectual disability (Curatolo et al., 2015; de Vries et al., 2015; Leclezio and de Vries, 2016), as evidenced by the WNV data collected in the current study. As such, there is also likely to be great heterogeneity in individual experiences of TSC. This variability could be reflected in the higher QOLIE-31-P domain meaningful change estimates compared to those previously generated in epilepsy populations (Wiebe et al., 2002; Cramer et al., 2004; Borghs et al., 2012).

### Limitations

Low sample sizes, specifically for the QOLIE-AD-48 and QOLIE-31-P, may have impacted the level of statistical power required for the known-groups analysis and the ability to detect change analysis. Therefore, we recommend reassessment of these properties in future studies. More generally, the low completion rates for the QOLIE-AD-48 and QOLIE-31-P may limit the generalization of the current findings to the TSC population. To this end, the high levels of missing data for the WNV underlines the real-life challenge of identifying suitable standardized measures of intelligence such as IQ tests for individuals across the full range of neurodevelopmental ability. Individuals with severe-to-profound intellectual disability may struggle to participate in and understand many formal measurements; therefore the assessment of functional abilities, such as through quantification of adaptive behaviors, may be a more appropriate alternative, resulting potentially in more complete data.

While the distribution-based methods used in this study have provided insight regarding the level of change that may be important, the limitations of the approach should be recognized. Distribution-based methods do not connect back to the patient experience of the disease, they are more related to scale precision and can generate inflated estimates (de Vet et al., 2006). Future research should seek to supplement the present results by conducting anchor-based analyses, which may aid decision making surrounding the most appropriate meaningful change estimate for each HRQL measure (de Vet et al., 2006).

## Conclusion

To our knowledge, this is the first evaluation using clinical trial data to establish the psychometric properties of the QOLCE, QOLIE-AD-48, and QOLIE-31-P for use in individuals with TSC, while there are some specific aspects across the measures that will require some further validation in future research. Importantly, we generated clinically meaningful change values specific to individuals with TSC. These findings should be useful for clinical practice and next steps in research.

## Author Contributions

All authors contributed to the study concept, design, manuscript editing, and final reviewing.

## Conflict of Interest Statement

PdV reports grants and honoraria from Novartis. All fees have been donated to non-profit organizations. DF reports salary support from Cincinnati Childrens Hospital for consulting work, research grants, and speaker honoraria and travel reimbursement from Novartis. PC reports personal fees for advisory boards from Eisai, Shire, Novartis, and speaker honoraria and travel reimbursement from Novartis. RN reports grants from EU (FP7), Shire, Zogenix, GW Pharma, and Eisai Medical Research, and personal fees from Eisai, Zogenix, and Novartis. MN is an employee of Novartis Pharmaceuticals Corporation. FH is an employee of Novartis Pharma AG. KS, EB and BB are employees of Adelphi Values and were commissioned by Novartis Pharmaceuticals Corporation to conduct this work. JL reports personal fees and research grants from Novartis.
